# Application of the Ostrom framework in the analysis of a social-ecological system with multiple resources in a marine protected area

**DOI:** 10.7717/peerj.7374

**Published:** 2019-08-14

**Authors:** Leopoldo E. Palomo, Alvaro Hernández-Flores

**Affiliations:** School of Natural Resources, Universidad Marista de Merida, Merida, Yucatan, Mexico

**Keywords:** Social-ecological system, Framework, Community-based governance, Coastal community, Multiple resources

## Abstract

The framework proposed by Ostrom (2009) has become one of the most utilized tools to address the complexity of social-ecological systems. Most cases use this framework to analyze the systems from the perspective of a single resource unit. However, the livelihoods in several coastal communities are diverse, so that the users interact with multiple common-pool resources, which makes their analysis difficult. In this sense, it is important to identify the key elements of management to achieve the sustainable use of the resources. In this study, we were able to do this in a coastal community where commercial fishing, ecotourism, and recreational fishing coexist. The system of interest, located in the state of Quintana Roo, Mexico, was subdivided by resource type using a multi-method approach to data collection including surveys, interviews, and records review. A conceptual map was developed that shows how the second-tier variables are integrated through the governance and actors with the biophysical system. The actors involved in lobster fishing achieved a more complex governance system, followed by the ecotourism and recreational fishing; the complexity of the governance was related with the equity level of the actors. The analysis revealed the research gaps to develop management strategies and improve the sustainability of the system.

## Introduction

The sustainable management of natural resources in marine protected areas (MPA), where fishing is the basis of the local economy, represents a challenge for decision-makers (Partelow & Boda, 2015; Perry et al., 2010). Different avenues have been proposed to address the complexity; however, the most used model in the last years is the social-ecological system framework (SESF) proposed by Ostrom (2009). The SESF of Elinor Ostrom has been broadly used as a diagnostic tool for the factors that contribute to the management of sustainable resources, in response to challenges presented in several case studies of human–environment interactions (Basurto, Gelcich & Ostrom, 2013; Nagendra & Ostrom, 2014; Partelow, 2015). Most of those case studies analyze the interactions between users (actors) who extract a single resource unit (RU, e.g., water, trees, lobsters) from the resource system (RS; e.g., parks, wildlife, hydrological systems) (Hinkel et al., 2015; McGinnis & Ostrom, 2014; Nagendra & Ostrom, 2014; Partelow & Boda, 2015). However, in some cases, the livelihoods of local people could be diverse (Allison & Ellis, 2001; Charles, 2012) where income comes from more than one resource (RU) belonging to a multiple resources system (MRS) whose interactions have not been broadly addressed. Given this context, our research question is: How can Ostrom’s framework be applied to identify the key elements of management and governance to achieve the sustainable use of multiple common-pool resources in a natural system within an MPA?

To this end, this article focuses on the application of Ostrom’s framework and modified by McGinnis & Ostrom (2014), to explicate a socio-ecological system, using as a case study a coastal community into a marine natural protected area, in which users perform more than one economic activity based on the natural resources. This research has the purpose of understanding how local users who obtain diverse benefits from the same MRS, implement different management strategies aimed at conserving the system and achieving the sustainable use of their resources at the same time. The problem that arises in most fishery literature is that it considers that income in these communities comes only from fishing, while it has been documented that some of these communities can obtain significant income from other non-fishing sources during periods of resource scarcity (Allison & Ellis, 2001; Charles, 2012; Phillips, 1998; Solares-Leal & Alvarez-Gil, 2005). It is precisely in this sense that the Ostrom’s framework can be used to understand the functioning of the economy in this type of community. The Caribbean village of Punta Allen in the Sian Ka’an Biosphere Reserve (SKBR), Mexico, is a community whose main activity is lobster fishing but there is a closed season for fishing of 4 months when fishers are involved in other livelihoods. In the last 20 years, fishermen have developed two other activities that compete economically with fishing: recreational fishing and ecotourism (Solares-Leal & Alvarez-Gil, 2005).

The Ostrom’s SES framework was used to update a diagnosis of the MRS, in which the information gaps are exposed, and the variables that enhance the sustainability and governance in the community are revealed. We believe that it is possible to monitor and evaluate the management and governance in a MRS using this framework to prioritize the actions in the decision-making process, to implement management strategies aimed at solving the problems of local users to improve their livelihoods.

## Background

In Mexico, the SESF has been sparsely used; Basurto, Gelcich & Ostrom (2013) applied the SESF to elucidate the factors that affect the self-organization capacity of fishers to avoid the tragedy of the open access regime. Lara Rivero & Hakizimana (2014) applied the SESF to classify the governance level in 31 cases of Mexican community forests by means of a meta-analysis. On the other hand, the application of the SES framework of Ostrom in scientific literature does not contemplate situations where a MRS is exploited in different ways by multiple users. In the real world, there are many cases in which people must diversify their livelihoods, particularly in fishing communities where season closures for fishing are common regulations, so they are not exclusively full-time fishers (Allison & Ellis, 2001; Charles, 2012; Gilliland, Sanchirico & Taylor, 2016). In the community of Punta Allen the locals are organized into different economic productive groups; however, the interest of most previous work in the bay focuses on the co-management system of lobster fishing based on the territorial use rights (Bennett et al., 2015; Cochran, 1998; Gutiérrez, Hilborn & Defeo, 2011; Solares-Leal & Alvarez-Gil, 2005; Sosa-Cordero, Liceaga-Correa & Seijo, 2008; Villanueva-Poot et al., 2017). More recently, tourism development in the SKBR has also attracted the interest of scholars, conducting studies on human–nature interactions and on the right-based coastal ecosystem (Arce-Ibarra et al., 2017; Solares-Leal & Alvarez-Gil, 2005; Villanueva-Poot et al., 2017).

This study is designed to contribute to knowledge in expansion of the application of SESF, toward effective conservation and management policies in the context of multiple resources and users at the local level.

## Study area

**Figure 1:**
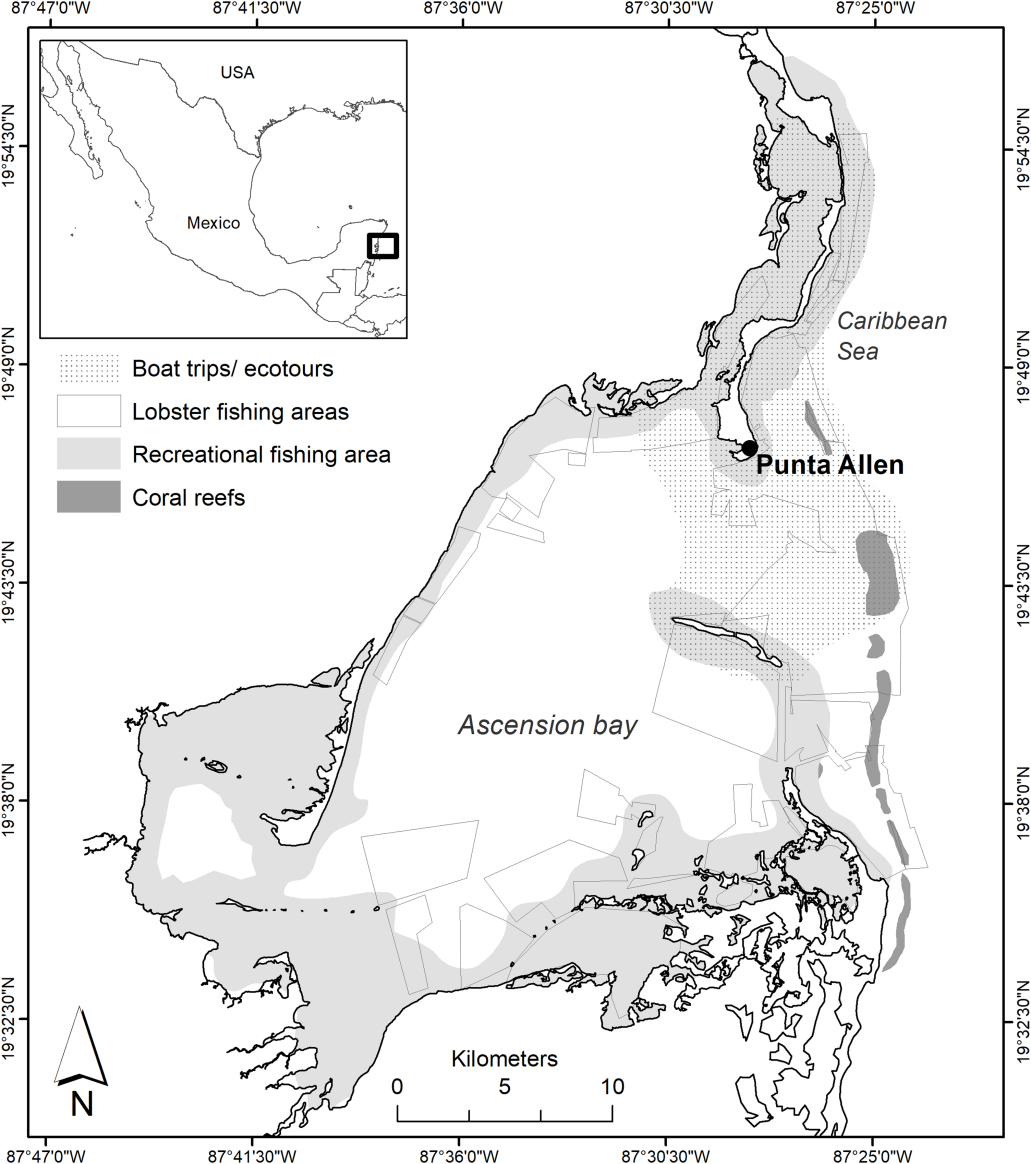
Area of study in Ascension Bay and operating sites of the economic activities of Punta Allen.

Ascension Bay is located at the south of the State of Quintana Roo, Mexico, in the SKBR. The bay has an extent of 740 km^2^, with shallow waters between one and six m deep. It is surrounded by dense mangroves forests and coastal dunes, and is protected from the front by the Mesoamerican Barrier Reef. Access to the bay by land is through a road along a narrow strip of sand of over 30 km of distance, which separates the coastal lagoon and the sea. At the end of the road there is a small fishers’ village known as “Punta Allen” (official name: Javier Rojo Gomez) (Fig. 1), which is located toward the north of the bay.

The village was founded by artisanal fishers of red lobster (*Panulirus argus*) in the middle of the 1950s. The most recent census indicates that the village had a population of 630 inhabitants in 2010, although throughout the year the number of visitors can be more than 40,000 tourists (Solares-Leal & Alvarez-Gil, 2005). The fishers are organized into a cooperative which obtained the exclusive concession to exploit the red lobster in the bay. With the growing tourist activity in the Mexican–Caribbean region, ecotourism emerged as an alternative livelihood in Punta Allen, and fishers created a tourist cooperative in 1994. Every year, more tourists come to Punta Allen in order to admire the sceneries, practice fly fishing, and snorkeling in the coral reefs.

## Material and Methods

The Ostrom framework was selected as the appropriate diagnostic tool to analyze the SES of the community because it provided organizational clarity and hierarchical structure and was conductive to future cross-site comparison. However, we realized that when considering the users and the variety of RUs in the same frame, it posed a huge challenge to analyze the complexity of interactions between users. Therefore, we decided to subdivide the SES into groups of users and in this way we were able to obtain valuable information on the economic, social, and environmental aspects of each activity, related to the rules of sustainable management at different scales of governance. This study used the framework of 56 second-tier variables published by McGinnis & Ostrom (2014) (Table 1) to characterize three sub-SES: (i) lobster fishery, (ii) ecotourism on boats, and (iii) recreational fly-fishing. The three sub-SES have geographic and temporal overlap and the local people can dedicate themselves to at least two of the three activities. Only few local fishermen have obtained licenses to carry out the three economic activities, so they have to hire another member of the community to operate their boats. The values and descriptors of the second-tier variables selected for the analysis were measured in field, taken from the literature or obtained from the community through semi-structured interviews, unstructured interviews, cooperatives logbooks, and participant observation during 12 three-day visits to the community, from March 2016 to November 2018, as summarized in Table 2.

**Table 1:** Second-tier variables of social-ecological system adapted from McGinnis & Ostrom (2014).

Social, economic and political settings (S) S1—Economic development*; S2—Demographic trends*; S3—Political stability; S4—Other governance systems; S5—Markets; S6—Media organizations*; S7—Technology	
Resource systems (RS)	Governance system (GS)
RS1—Sectors	GS1—Government organizations
RS2—Clarity of system boundaries	GS2—Nongovernment organizations
RS3—Size of resource system	GS3—Network structure
RS4—Human-constructed facilities	GS4—Property-rights systems
RS5—Productivity of system*	GS5—Operational-choice rules
RS6—Equilibrium properties*	GS6—Collective-choice rules
RS7—Predictability of system dynamics*	GS7—Constitutional-choice rules*
RS8—Storage characteristics	GS8—Monitoring and sanctioning rules
RS9—Location	
	Resource units (RU)
Actors (A)	RU1—Resource unit mobility
A1—Number of relevant actors	RU2—Growth or replacement rate*
A2—Socioeconomic attributes*	RU3—Interaction among resource units
A3—History or past experiences	RU4—Economic value
A4—Location	RU5—Number of units*
A5—Leadership/entrepreneurship	RU6—Distinctive characteristics
A6—Norms (trust-reciprocity)/social capital	RU7—Spatial and temporal distribution*
A7—Knowledge of SES/mental models	
A8—Importance of resource (dependence)	Related ecosystems (ECO)
A9—Technologies available	ECO1—Climate patterns
	ECO2—Pollution patterns*
	ECO3—Flows into and out of focal SES
Action situations: Interactions (I) → Outcomes (O) I1—Harvesting; I2—Information sharing; I3—Deliberation processes; I4—Conflicts; I5—Investment activities; I6—Lobbying activities; I7—Self-organizing activities; I8—Networking activities; I9—Monitoring activities*; I10—Evaluative activities*; O1—Social performance measures; O2—Ecological performance measures*; O3—Externalities to other SESs	

**Note:**

* The information on these variables is partial or does not exist; These gaps are described throughout the narrative text.

**Table 2:** Source of data collected for the SES framework of the multiple resource system of the Ascension Bay.

Type of data	Lobster fishery	Ecotourism services	Fly fishing
Logbooks data	Cooperative logbooks. Historic catch data, period: 2007–2017	Partial month data from two main touristic cooperatives since 2015–2017	Partial month data from three fishing lodges and cooperatives since 2015–2017
Interviews with local leaders	Unstructured interviews with four of the cooperative leaders	Unstructured interviews with three of the cooperatives leaders	Unstructured interviews with eight of the most expert local guides
Surveys	33 fishers	18 local guides	27 local guides and 92 surveys to foreign anglers
Spatial boundaries of the operation areas	Fishing areas. TURF’s map (Headley et al., 2017)	Marked on a map by local guides	Fishing area obtained from 54 trajectory records using GPS on boats

The conceptual framework of a SES proposed by Ostrom (2009) and later modified by McGinnis & Ostrom (2014) is composed of eight first-level variables: The RS and the RUs that complement each other in the natural component; the governance system (GS) and the actors (A) that constitute the social component. These components interact through a focal action situation to produce the interactions (I) and outcomes (O) from either natural or social components. Therefore, the study followed the three-step process of McGinnis & Ostrom (2014) for the characterization of a SES framework:

1. The focal level of the analysis that describes the interactions between the users with the products and services in a particular GS. In Ascension Bay, there are three main economic activities based on the use of natural resources. One is the red lobster fishing; the other two activities are based on tourism, which are recreational fly-fishing and ecotourism by boat. Given that each activity has specific RUs, users (actors), rules (governance), interactions, and outcomes, the three activities were separated into three sub-systems (sub-SES), but that are actually integrated both at the user level and in the biophysical system.
2. Select the second-tier variables that will be measured within the three sub-SESs, designed to unravel the SES framework for each actor (Table 1).
3. Establishment of a clear SES framework to compare its results with other studies or disciplines, and with the intention of generating a mutual benefit from exchanging information. In this regard, the selection of the second-tier variables was made in accordance with each of the three sub-SES already described; the adaptive strategies are analyzed in the light of the model of community-based governance.

One of the main challenges to integrate this study was the data collection. Although fishing and tourism activities have guidelines and regulations that encourage them to maintain up-to-date records, the institutional capacity of the government is exceeded due to the insufficient manpower and financial resources. For this reason, the records are incomplete or simply non-existent. These data were organized, summarized and supplemented with additional data obtained through surveys and interviews (Table 2). The Ascension Bay SES diagnosis was developed following the Ostrom’s framework and is presented in a narrative style in three sections and subsequently, a nested conceptual map of the three sub-SES is developed.

## Results

In this section, each of the three socioecological subsystems are described with the first and second-tier variables. The order in which are described is: (1) The social-ecological subsystem of lobster fishery, (2) The social-ecological subsystem of boat ecotourism, and (3) The social-ecological subsystem of recreational fly-fishing.

### The social-ecological subsystem of lobster fishery

The actors [A1] are the members of the cooperative who hold the concession for catching lobster in the bay since 1994. In 2014 the cooperative had 42 members and 48 boats (Headley et al., 2017). [A3] The human settlement of Punta Allen dates back to the 1950s with the arrival of agricultural workers. After agricultural activities decreased in the late 1960s due to coconut lethal yellowing disease, people focused exclusively on the lobster fishery (*Panulirus argus*).

[A4] In 1968 the fishers’ village was established at the end of a sandbar north of the mouth of the bay (Fig. 1). In that year, the fishing cooperative “Vigia Chico” was created. [A5] Before the concession were granted to the cooperative, fishers had internal agreements to distribute fishing grounds, so historically they have established inheritable or transferable territorial rights (Seijo, 1993). [A6] In 1988, the cooperative administration established new and stricter internal rules for its members, such as severe sanctions for the illegal sale of lobster or fishing in the grounds of other members, which led to the expulsion of some members of the cooperative with a subsequent decrease in the membership from 120 to 70 (Méndez-Medina, Schmook & McCandless, 2015). [A9] The fishing technique is an artificial shelter for lobster, locally known as “casita” made of steel wire covered with concrete. To catch live lobster, fishers only have to dive (apnea) and use the nets to extract the lobster from the shelters. There is an estimate of 27,000 artificial shelters settled in the bay (Headley et al., 2017; Villanueva-Poot et al., 2017). With the support of GPS, each fisher knows and have perfect control of the exploitation of his own shelters.

[RS1] The RS of the lobster fishery is comprised of all the elements of the environment and marine ecosystem that supports the lobster population and the fishing grounds (Fig. 1). [RS2] Currently there are 69 lobster fishing grounds (locally known as “campos”). The fishing grounds are clearly delimited in a geographic information system developed and donated by an academic institution (Villanueva-Poot et al., 2017). [RS3] The fishing grounds occupy 30% of the bay (170 km^2^), and are located mainly near the mouth, near coral reef formations, and in the mangrove forests.

[RS4] In the village, the cooperative has constructed a dock, office, freezers, storage rooms, and an auditorium. [RS5] The natural system is highly productive as it has large areas [RS9] of seagrass beds, mangroves and coral reefs, which support a great biodiversity, such as fish, sea turtles, sea birds, and dolphins, among other taxa. [RS6] Due to the relatively undisturbed interface of sea and land along a well-conserved coastline, SKBR was declared a World Heritage Site in 1987 (UNESCO, 1988). [RS7] The dynamics of the ecosystem can be predicted to a certain extent, as more information is produced thanks to the empirical knowledge of the fishers.

[RU1] The lobsters are the RUs, since they are the target species. Because the catch depends mainly on the artificial shelters and these allow for the management of the lobsters in there, only the lobsters that were inside the artificial shelters were considered as RUs for analytical purposes. [RU3] The interaction between the fishing grounds can be negligible, since lobsters tend to stay in the same place. [RU4] The historical data series based on the cooperative’s logbooks, indicates that from 2007 to 2016 the annual average catch was 75 tons (S.D. = ±19.7) [RU5], with an estimated value of US$1.05 million per year. [RU6] The spiny lobster forms a meta-population throughout the Caribbean Sea, that is, a geographically separated population in different stocks or sub-populations connected through some stages of the life cycle, such as larval dispersal or adult migration. The Ascension bay holds one of those sub-populations.

[GS1] At the national level, the National Commission for Fisheries and Aquaculture (CONAPESCA) is the federal agency that regulates the fishing resources and has granted the concession to exploit the lobster in the bay to the Vigia Chico Cooperative. In addition, the Harbor Master’s Authority has issued navigation licenses to their fishing boats. Because the bay is within a MPA, additional restrictions for lobster fishery has been established by the SKBR Management Program (SEMARNAT-CONANP, 2014), endorsed by the National Commission of Natural Protected Areas (CONANP) and enforced by the Federal Attorney for Environmental Protection (PROFEPA). [GS2] Since the foundation of the SKBR more than 50 institutions and non-governmental organizations have participated in scientific research, projects to conserve the natural resources, environmental monitoring, and support to local communities (SEMARNAT-CONANP, 2014).

[GS4] At the local level of governance, fishers from Punta Allen have developed property-rights system based on cooperation, which grants exclusive fishing rights of specific parcels or “campos” to each member of the cooperative (Seijo, 1993; Solares-Leal & Alvarez-Gil, 2005; Sosa-Cordero, Liceaga-Correa & Seijo, 2008). [GS5] Federal regulations allow the use of traps, command fishers to release undersized lobsters and berried females. There is a closed season for fishing from March 1 to June 30 (DOF, 2016). [GS6] The internal rules of the cooperative are based on democratic procedures of election. [GS7] The GS at local level is supported by the General Law of Sustainable Fisheries and Aquaculture. [GS8] Sanctions at the federal level are enforced by CONAPESCA through periodic visits of officers to the area.

[I1] The lobster stock is harvested at sustained levels (Sosa-Cordero, Liceaga-Correa & Seijo, 2008; Villanueva-Poot et al., 2017). [I2 and I7] This and other information (e.g., financial reports or earlier closure seasons due to adverse weather conditions) is shared in periodical meetings or assemblies (Sosa-Cordero, Liceaga-Correa & Seijo, 2008). [I3] Cooperative members have established democratic and accountable deliberative processes, and [I4] internal conflicts are resolved in the assembly. [I6] The leaders participate actively in the Confederation of Fishing Cooperatives of Quintana Roo. [I8] Frequently, cooperative leaders establish commercial networks with local seafood markets such as Cancun and with buyers from other cities, such as Merida. [I5] The artificial shelters imply a large investment, that is why it is a decision of the fishers to obtain more fishing grounds. [I9] The cooperative maintains reliable records on individual production per day, fishing ground and the number of artificial shelters surveyed; [I10] in addition, it makes empirical biological evaluation of the state of lobster that supports decision-making processes.

[O1] The social performance of this activity is high; for instance, Villanueva-Poot et al. (2017) mention that the income distribution among the fishers of the cooperative achieved a high level of equity and solidarity compared to other fisheries. [O2] In addition to the fishing mortality produced in the lobster population, the ecological performance on the impact of the shelters deployed in the bay has not been studied; however, [O3] there is a positive outcome of the artificial shelters in the fishing grounds as these present a rapid colonization generating new habitats, causing a positive externality on the benthic species including those of interest for recreational fishing.

[ECO1] Historically, this area has been affected by several hurricanes, with an adverse influence on human settlements. On average, every 2.5 years, a hurricane hits the area. Because strong winds bring down houses and infrastructure, human settlements are vulnerable, but the ecosystems have the ability to recover from the disturbances maintaining a good conservation status (SEMARNAT-CONANP, 2014). The excess rains or hurricanes also affect lobster production. [ECO3] Because spiny lobster forms a meta-population, it is considered a straddling stock for the Caribbean countries by the Law of the Sea. In this regard, the management strategies of Ascension Bay favors the increase of the refuge area for the lobsters and the sustainability of the fishery.

[S1] Lobster fishing represents the main livelihood in the community. [S3] The democratic values of the fishers can help the cooperative to adapt to future situations and maintain the equity of the benefits and political stability. [S5] Market is the most important driver of the fishery. In general, there is good demand and price for the lobster (it can vary from US$20 to US$25 kg of tail). [S6] The leaders are authorized to make declarations to the media about the situation of the cooperative.

### The social-ecological subsystem of boat ecotourism

The actors [A1] are the tourists and the certified naturist guides. In total there are 85 guides members. This study considered data from two cooperatives (“Vigia Grande” and “Punta Allen”). Based on a data series from 2015 to 2017, we estimated a total of 40,945 tourists who practiced snorkeling, watching bird, dolphins, and sea turtles in their natural habitat (Solares-Leal & Alvarez-Gil, 2005; Sosa-Cordero, Liceaga-Correa & Seijo, 2008). [A3] Since 1986 some tourist activity was observed. To respond to the increase in demand for tourist services, the government encouraged the creation of the first tourist cooperative in 1994. Subsequently, three other cooperatives were organized, [A5] which gave the locals an effective system by which to organize the activity. [A6] The social capital facilitated the villagers to develop and agree upon a series of rules and practices to carry out ecological tourism aligned with the federal regulations. In general, the rules are well implemented.

[A7] Over time, the actors have learned about the importance and vulnerability of ecosystems and have acquired a high sense of conservation and responsibility. [A8] Ecotourism has acquired relevance in the community, involving a large part of the population employing naturist guides and other indirect jobs like restaurant services, handcrafts, and lodging services. [A9] The cooperatives have 42 authorized boats adapted to provide tourism services.

[RS1] The sector is tourism and the RS is represented by all the ecosystems of the visited places (Fig. 1). [RS2] The boundaries where the activity takes place are well defined, as well as the routes that tour guides follow. [RS3] The RS coverage is 203 km^2^ (Fig. 1). [RS4] The infrastructure to provide the service consists of an office to receive tourists and a pier dedicated to the activity. [RS5] The landscapes in the bay attract a large number of tourists, so these sites can be considered highly productive in terms of tourism. [RS6] Some studies indicate that in general, coral reef ecosystems and their biodiversity present a good state of conservation (Núñez-Lara, Arias-González & Legendre, 2005; McField et al., 2018; Arias-Gonzalez, 2012). [RS7] It is necessary to study the dynamics of the ecosystem to be predicted. [RS9] The coral reef formations, channels, and islets in the bay are clearly localized.

[RU1] This is a non-extractive activity; therefore, the RUs are the individual ecosystems visited, including inland water channels through the mangroves, islands around the bay, coral reefs, and sandy beaches. [RU2] The biodiversity plays an important role in maintaining the health of these ecosystems. [RU3] The interactions among RUs are very strong, since all the ecosystems in the bay are interconnected through different biophysical elements (e.g., mass of water, fish, mollusks, vegetation, etc.); connectivity can improve the resilience of the ecosystems (Resilience Alliance, 2010).

[RU4] The economic value considered in the study was the total annual income for the tourist cooperatives that reached US$923,000. [RU5] In practical terms, the number of units is the number of sites visited most often, which are 14 coral reef formations. [RU6] The most distinctive characteristic of these ecosystems is that they are relatively isolated from human influence.

[GS1] At the national level of governance, the federal agency in charge of natural protected areas is CONANP, which establishes the control measures of ecotourism activities in the area. The boats must be registered in the Harbor Master Office. It is strictly enforced that all boats belong to any of the four cooperatives, which represent the local level of governance. [GS3] Locals have developed strong social networks, market networks, and environmental networks. The tourism cooperatives have created alliances to negotiate and standardize the prices to avoid conflicts. [GS4] Tourism cooperatives are the only ones authorized to provide the service, and they have developed a system to equitably assign the number of visitors.

[GS5] Tourism activities take place all year round. The activities of boat riding and free diving are subject to a maximum of six visitors per boat. To reduce erosion in the channels and lagoons, the boats are allowed engines up to 40HP. It is mandated that before the visit, tour operators give prior information on the number of visitors they are bringing, so the tourist cooperatives can program and prepare the boats that will provide the service. The maximum number of boats allowed is limited to 140. At the local level of governance, cooperatives have established additional measures to protect both tourists and the ecosystem: well-defined navigation routes to avoid collision of vessels. Speed limits to avoid collisions and avoid damaging the coastal dunes. Departure times of at least 20 min apart from one boat to another (SEMARNAT-CONANP, 2014).

[GS6] Collective choices rules are developed in cooperatives in democratic processes. The self-organization is very important due to the relatively large number of visitors that everyday arrive at the town; in peak days, cooperatives can receive up to 250 tourists, which represents 38% of the local population. [GS7] The rules are properly endorsed by the SKBR Management Program (SEMARNAT-CONANP, 2014). [GS8] To verify compliance, federal officials of CONANP and PROFEPA make regular visits to the site.

[I1] The main interaction between the visitors and the natural system, is the entrance of the latter to the ecosystems for the scenic enjoyment. In the site, there is an estimate of 6,824 trips per year with no studies on the maximum acceptable. [I2] Cooperatives exchange information regarding the availability of boats for tour operators everyday to coordinate the visits. [I3] Each cooperative has its own deliberative processes (assemblies), and sometimes, the leaders hold informal meetings to discuss relevant operational issues. [I4] There are minimum conflicts between the members of the cooperatives, which can be resolved at their assemblies.

Other interactions are the investment of cooperatives to preserve the ecosystem [I5] by training their tour guides in better practices (endorsed by the Ministry of Tourism) and in the maintenance of equipment and infrastructure. [I6] Some lobbying activities of the cooperatives leaders can occur when the collective interests are hampered. [I7] Each cooperative organizes periodical assemblies to take internal agreements, or share financial reports to their members. [I8] The marketing network of tourist services at state and community level is strong and competes with other destinations in the Mayan Riviera. The tourist cooperatives have established commercial contracts and alliances with tourist operators of the Mayan Riviera and Tulum. [I9] The four cooperatives maintain records on the number of tourist and trips per day. [I10] There is a basic evaluation of the climatic conditions for the decision-making processes regarding the appropriate routes and sites.

[O1] The social performance of this activity is good, since it is the activity with the highest rate of increase and with a high economic spill in other sectors of the economy, with an even distribution of benefits among the participants. [O2] In terms of ecological performance, the impact of divers on coral reefs and other ecosystems has not been assessed, as the number of visitors is around 40,000 per year. [O3] This activity has negative externalities to recreational fishing, due to the use of spaces near to the recreational fishing sites where the overcrowding of tourists causes disturbances in the target species.

[ECO1] Atypical massive quantities of pelagic macroalgae (*Sargassum fluitans*) in recent years into the entire Caribbean coast have negatively affected the tourist industry (Rodríguez-Martínez & Van Tussenbroek, 2017). One year after the Hurricane Gilbert hit Quintana Roo in 1988, a mass bleaching event occurred in the coral reefs of Isla Mujeres, Puerto Morelos, Mahahual, and Sian Ka’an; however, the mortality was moderate to low in the different seasons when there were increases in temperature at sea, with subsequent slow recoveries (Gardner et al., 2003; Reaser, Pomerance & Thomas, 2000; Wilkinson & Souter, 2008). This is latent in Sian Ka’an, as there are reports showing that around 47% of coral reefs in the Mexican Caribbean suffered bleaching in 2015 and 2016 with low mortality (McField et al., 2018). Likewise, hurricanes and storms can destroy coral reefs which then become vulnerable (Reaser, Pomerance & Thomas, 2000). Regarding the ocean acidification, different authors mention that the calcification rates of corals can be affected, depending on the species, but in general most of them can tolerate the expected pH reduction (Crook et al., 2012; Pandolfi et al., 2011).

[ECO2] There are no studies about the impact of boating on the coral ecosystems. The impacts could occur with the increased number of tourists in the natural areas. Additional research is needed on the impact of the waste systems and water extraction in the area of study. [ECO3] The coral reef ecosystems of Ascension Bay belong to the Mesoamerican reef system, therefore, the mass of water and organisms flow to and from that system. For this reason, it is necessary to carry out more studies where tourism is practiced.

[S1] With the growing economic development in the area, ecotourism in Punta Allen has become the second most important livelihood for the community. [S2] According to figures from CONANP, from 2007 to 2015 the number of visitors to the SKBR were 80,000 per year, this was twice the number observed before 2005. [S3] This trend represents a challenge for the community, so according to interviews with villagers who have been able to resolve past conflicts, the democratic values of the cooperatives provide political stability to the village. [S5] Nevertheless, the actors must be clear that the activity is subject to the influence of local and international markets, [S6] as tour operators advertise the site on websites to attract a greater number of visitors, and local cooperatives advertise their services in social networks.

### The social-ecological subsystem of recreational fly-fishing

[A1] For the purposes of this study, the actors are considered the anglers and the fishing guides. There are 65 local fly fishing guides. There are no records on the exact number of anglers, but from interviews with fishing guides and lodge managers, we estimated that between 800 and 1,200 anglers visited Punta Allen per year (average = 1,143 anglers per year).

[A3] Since the 1980s, foreigner anglers had contacted local fishers to came to Punta Allen in search of pristine ecosystems to practice fly-fishing sporadically. As the activity gained prestige throughout the world, in 2015 Punta Allen received the nickname of “The Permit Capital of the World.” This activity can be considered as the most recent livelihood in the community. [A4] Most of the fishing guides are locals and few of them are from other communities. From our surveys, 98% of the anglers came from the US.

[A5] The fishing guides are not organized in formal groups, but some of them take the leadership to make independent agreements with the lodges to establish the prices of the contracts, and this information is disseminated. [A6] We observed that the experience of the fishing guides and their ability to interact with the anglers is their social capital, since it is a very important aspect that motivates the decision of the anglers to visit the site. All fishing guides carry out other activities and some of them are members of the tourist cooperatives and/or the lobster fishing cooperative. [A9] Currently, there are 65 boats authorized to provide the service, of which 33 belong to cooperatives and 32 to independent individuals, including foreigner businessmen. A total of 20 of those boats have concessions to provide both fly fishing and ecotourism service. The boats are adapted to sail in shallow waters (60 cm deep), with the capacity to carry up to two anglers and two local guides.

[RS2] The fly fishing sites are well defined (Fig. 1) and [RS3] the effective fishing area is 265 km^2^, estimated through the monitoring of 53 fly fishing trips between January 2016 and September 2017. [RS4] There are ten fishing lodges with 64 rooms for a capacity of up to 117 anglers, but many of them only operate from 4 to 6 months per year. [RS6] According to our survey, 88% of the anglers answered that the ecosystem presents a good state of conservation compared to other sites. [RS9] The ecosystems where the activity takes place, are located mostly in the “flats” of the bay, which are areas of shallow water (<0.60 m depth), like marshes, sites near the mangroves and sea grasses (Fig. 1).

[RU1] The RUs are the four targeted species that are: bonefish (*Albula vulpes*), tarpon (*Megalops atlanticus*), permit (*Trachinotus falcatus*), and snook (*Centropomus undecimalis*). [RU2] Although these species move freely in the interior of the bay, the migratory, and recruitment processes of the different populations have not been documented in this area. Ascension Bay has abundant mangroves that serve as nursery areas for recruits of these and other species. [RU3] Because the authorized fishing is catch and release, it is not considered an extractive activity, and fishing mortality is presumed to be low, although there are no specific studies on it. Adams (2017) reports a mortality of 5% of these species (permit, bonefish, snook, tarpon) captured in other sites with catch and release. In this sense, there are no evidence that shows a decline of the targeted species populations. [RU7] The spatial distribution of the four species is heterogeneous according to their habitat preferences.

[RU4] The average price to rent a boat was estimated at US$180 per day, in 2016. The total income that the local fishing guides got in that year was estimated to be US$737,450. [RU6] The presence of multiple sport fish in the same place is considered by anglers a distinctive characteristic, since they can achieve a “grand slam” (three species in 1 day) and/or a “super grand slam” (four species in 1 day).

[GS1] At the federal level, the GS is represented by CONANP, which has established the recreational fishing rules in the modality of catch and release. In addition, the Harbor Master Office issues concessions to the boats to provide services of fly-fishing. As for the anglers and guides, they have to get fishing licenses from CONAPESCA. [GS2] The activity is supported by different non-governmental organizations like Bonefish and Tarpon Trust, Yellow Dog, and Amigos de Sian Ka’an. [GS7] The activity is properly authorized in the General Law on Sustainable Fisheries and Aquaculture, through the norm NOM-017-PESC-1994 and the SKBR Management Program. [GS8] Federal officials of CONANP and PROFEPA make regular visits to verify operations.

[GS3] In general, the fishing guides have an internal social network. The groups only participate in providing the services and the lodges control the market networks, and environmental networks. [GS4] Because the fly fishing concession can be granted to any boat (private local and non-local), the local guides declared that this type of regime generates uncertainty, since they perceive it as a threat to their livelihoods, with fear that owners of the private lodges may not need their services. [GS5] There are few federal regulations to practice the activity, like catch and release and have the proper documents to perform the activity. [GS6] In 2013, the local actors agreed to implement a Code of Conduct, which is binding for boat owners and fishing guides. The purpose of the code was to implement better catch-and-release fishing, and anglers reported full compliance with the code, which represents a social capital for the local guides [A6]. Some rules were: Do not invade areas for recreational fishing already occupied; use artificial lures; do not use fishing nets.

[I1] This is a non-extractive activity, but the interactions can be measured as the number of anglers that visit the area. We estimated that the 1,143 anglers that visited the bay performed a total of 3,430 fishing trips in 2017. [I2] There is limited information shared among small groups of fishing guides. [I3] Each group of fishing guides or individual guides have their own interest, but sometimes they meet to establish non-binding general agreements with minimal deliberative process. [I4] There are few conflicts between lodge managers and fishing guides due to disagreements in the prices. On peak days there are conflicts with ecotourism boats due to overcrowding. Some conflicts between guides for non-compliance with the code of conduct.

[I5] Cooperatives and guides invest in training for certification as tourist guides (endorsed by the Ministry of Tourism). Owners of the lodges organize fishing tournaments and part of the profits are earmarked for community assistance.

[I6] Lobbying is limited as there are no organized formal groups. [I7] The organization between guides is at two levels: (1) between leaders of different groups, and (2) in the groups, which facilitates the achievement of collective agreements. [I8] Networking activities are carried out through the lodges and international fishing clubs and some lodges attend international fairs to promote the site. [I9] There are no systematized records for all recreational activities related to fishing, including those of the lodges. Additional research is needed on the target species in the bay.

[O1] The activity provides great benefits to an important sector of the community, in particular to families of fishing guides, and other activities such as restaurants and housekeeping in the lodges. [O2] According to the interviews, the anglers stated that the quality of the fishing is good. The catch of target species has an impact on fish mortality by release, but it has not been estimated. However, the community contributes to the conservation and protection of marine life inside of the bay by avoiding commercial fishing with the exception of lobster.

[O3] Although the flats are located in lobster fishing areas or ecotourism sites, the guides look for quiet and isolated places, so this activity is considered low impact to the environment and, according to the recreational fishing guides, the populations of targeted species receive a positive externality from the artificial shelters for lobsters.

[ECO1] The abundance of target species is susceptible to changing weather patterns, such as variations in wind patterns, salinity due to drought patterns or heavy rainfall. [ECO2] As long as CONANP controls lodges infrastructure development in the area, and the human population does not increase, these threats can be considered low. [ECO3] Fishing guides mentioned that outside the bay the targeted species are captured for commercial purposes with nets, which reduce the benefits of the activity.

[S5] The recreational fishing service starts with the promotion of local lodges in international fishing clubs that offer all-inclusive packages from 3 to 6 or more days of fishing in the bay. This activity is in great demand by foreign anglers from developed countries (mostly from the US) who travel to different Caribbean countries, like Cuba, Belize, Honduras, or Mexico, in search of these trophy species (Adams & Cooke, 2015; Arlinghaus & Cooke, 2009; Barnett et al., 2016). Because quality of the service and target population are important in the market, Punta Allen compete with those destinations. [S6] Fishing clubs, lodges, and international organizations advertise the site on websites to attract foreign anglers.

### Toward a nested conceptual map of the three sub-SES of Ascension Bay

A segmented vision of the MRS using the SESF is useful to understand how the whole system functions. For a holistic understanding, we must consider the multiple interactions of each of the parties in a multi-level environment (i.e., biophysical and social). This new approach of the SES framework can be represented in a conceptual model of the system as a whole. We start with the biophysical bases common to the three sub-systems, i.e., the RS of Ascension Bay, which comprises all the elements of the environment and marine ecosystems that supports the lobster fishery, the fly-fishing, and the ecotourism. It includes areas of seagrass beds, mangroves, coral reefs, and flats, which support a great biodiversity of crustaceans, fish, mollusks, echinoderms, sea turtles, sea birds and dolphins, and other. Therefore, the RS represents the bases of the three sub-SES.

**Figure 2:**
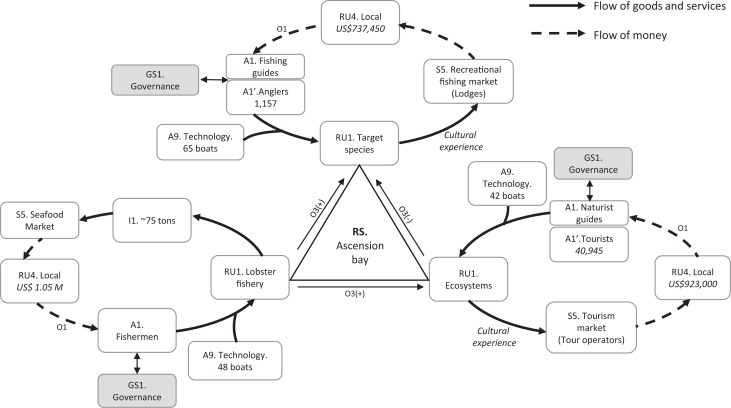
Conceptual map of the three sub-SES of Ascension Bay.

The RS provides the RUs [RU1] (lobster, target fishes, and landscapes) to the human component [A1]. The actors employ technologies [A9] (e.g., boats, artificial shelters) to extract or access the resources. As actors interact with the RUs, which become the central part or raison d’être of the users, these interactions result in the production of goods and services. Because lobster fishing is the only extractive activity in Ascension Bay, harvesting [I1] is important for the conceptual model, contrary to tourism activities, in which case services are cultural experiences. The next step in the conceptual model is to identify the market forces [S5] that drive the activities; in our case, these are the national market for seafood and the international tourism markets. As a result of the exchanges in the markets, the community obtains income from the economic value [RU4] of these goods and services. Once the locals receive the corresponding payments, the cycle is closed, so these interactions can be represented as a circular process that can be replicated infinitely, as long as the same conditions are maintained (Fig. 2).

The outcomes of these interactions differ according to the level of organization of the local actors; for example, income from lobster fishing and ecotourism is distributed more equitably through cooperatives [O1], while income distribution in fly-fishing varies among fishing guides. The actors use resources that impact the ecological system generating externalities in the related SES, for example they could affect the productivity and affect the ecosystems (Binder et al., 2013; Charles, 2012; Perry et al., 2010). On the other hand, lobster fishing has positive externalities [O3] in the other two activities, since artificial shelters enhance the habitat for marine life, while ecotourism generates negative externalities to recreational fishing since visitors disturb the fishing sites.

**Figure 3:**
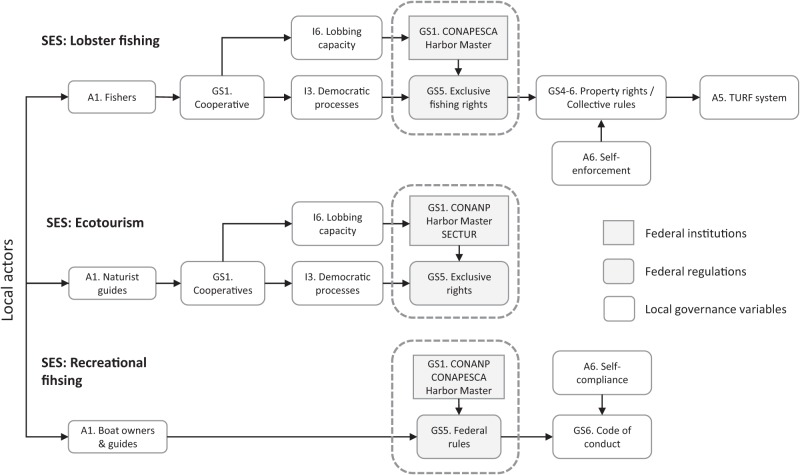
Diagram of the structure of the governance systems for the three sub-SES.

With the second-tier input–output variables a nested conceptual model was created, which shows the links between the variables. Nevertheless, there is an additional element that determines the functioning of the MRS: the governance, which in our model, is directly related to the actors. Due to the complexity of the GS, we decided to separate these into independent models according to the SES (Fig. 3).

In the lobster fishery, the GS has a more complex structure involving eight second-tier variables. At the local level, the governance is represented by the cooperative [GS1], which has democratic deliberation processes through the assembly [I3]. At the same time, the cooperative leaders have a strong lobbying capacity [I6] to negotiate with governmental (e.g., CONAPESCA and CONANP) and non-governmental agencies. When the federal government grants the cooperative legitimate exclusive fishing rights in the area [GS5], fishers enact additional collective rules [GS4–6] that grant them individual property rights due to their social capital for self-enforcement [A6]. The individual fishing grounds (“campos”) are managed under the approach of territorial use rights for fishing system (TURF) [A5].

In ecotourism, the natural guides and local boat owners are organized in cooperatives [GS1]. These cooperatives have deliberation processes [I3] and their leaders have the capacity to lobby [I6] with the federal institutions (CONANP and Harbor Master), which grant boat licenses exclusively to the community and the issued tourist guide credential by the Ministry of Tourism (SECTUR) [GS5]. Recreational fishing has the simplest GS, with only four identified variables. The boat owners [A1] that acquire the licenses for recreational fishing may belong to ecotourism cooperatives or be independent, for that reason the administrative procedures vary. The federal government (CONAPESCA, CONANP, and Harbor Master) grants licenses and establishes specific regulations for recreational fishing, such as capture and release [GS1-5]. In addition, the fishing guides with the support of non-governmental organizations and the federal government developed the Code of Conduct for Better Fishing Practices [GS6] with strong self-compliance [A6].

The users who possess the property rights of the resources can become owners, proprietors, claimants, or simply authorized users (Schlager & Ostrom, 1992) depending on the GS. In the case of the lobster fishery and ecotourism, the fishers and guides could be considered as proprietors, since they have right to access, withdraw, manage and exclude other potential users. For the fly-fishing, the actors are just authorized users, but are not allowed to withdraw any fish from the natural system.

## Discussion

The relevance of a SES model is that, in a broad sense, it connects tangible and relevant problems of the resources that are used with the decision-making processes at the local level, which can improve the GS (Charles, 2012). Ostrom’s SESF, being a useful as a diagnostic tool at all hierarchical levels (Binder et al., 2013; Partelow, 2015), was applied to accomplish our interest in analysis of Ascension Bay as a case study on a local scale, and to explain the sustainable outcomes considering the multiple uses in the bay. To our knowledge, there are no studies that analyze how multiple RUs and actors connect in a single SES framework, hence, the application of Ostrom’s SFSF was a challenge to achieve a holistic analysis in the present study. The interactions and outcomes of each sub-SES were fundamental in identifying the structure and functioning of the RS to recognize the key elements of governance, which can be useful to improve management programs (Berkes & Seixas, 2005); Although the general framework of Ostrom allows the incorporation of additional third-tier variables to obtain more details, in our case the second-tier variables were sufficient to generate a conceptual model that can be compared with other research (McGinnis & Ostrom, 2014).

The Ascension Bay as RS is the core of the SES, which provides the basis to maintain the three activities. The actors use technologies either to extract lobsters or to allow tourists or anglers access the RS. The harvest levels or the number of tourists and anglers that visit the bay will determine the sustainability of the biophysical system as a result of their impact on it. As the system recovers to acceptable levels, it will remain resilient. Therefore, the economic value of the resources will incentivize the local actors to make efforts to conserve the resources (Gutiérrez, Hilborn & Defeo, 2011). This mean that they have empirical knowledge or mental models of the SES. The operating processes of economic activities, driven by market forces, are controlled by a government scheme where the integration of local and federal government levels perform different but complementary functions.

The three activities are managed under different local GSs that resulted in different outcomes for the entire system. In the GS, the activities in which local actors organized themselves into cooperatives (i.e., lobster fishing and ecotourism) had greater capacity to lobby with the federal government. These activities showed the development of collective-choice rules accompanied by self-enforcement. Although the recreational fishing GS lacks formally organized groups, there is a code of conduct that derived from collective-choice rules and self-compliance. The social resilience depends in great part on social capital (Resilience Alliance, 2010), that in this case comprises organizational systems, self-enforcement, and collective-choice rules. Democratic values and equity are very important to maintain the coexistence and cohesion of the local society network because most of the people are relatives and neighbors. To strengthen the social network, it is essential that local rules of collective-choice be supported and aligned with federal rules, otherwise, lack of trust in the rules may hinder the social network. The social outcome has been an adaptive process in the coastal community of Punta Allen that resulted from natural disasters, social, economic and political settings, and self-determination, system that has evolved over time. Similar scenarios have been observed in some indigenous communities (Walker et al., 2004) that have evolved an improvement in resilience, adaptive management, and systems in multiple equilibria.

Charles (2012), mentions that the local GS is frequently embedded in a larger spatial scale, so that a community perspective provides relevant information on the social-ecological system. On this regard, we found different governance arrangements in the community, with the lobster fishery being the most complex. The GS in ecotourism had agreements that were widely applied and allowed to harmonize the activity and reduce conflicts, but still lack larger-scale agreements to measure and prevent ecological impacts. Recreational fishing was an activity that seemed to have a less developed GS, and involved fewer community members relative to the other resources subsystems. However, Punta Allen is nowadays a main destination for recreational fishing at a level similar to that seen in Cuba, Belize, Honduras, or the Florida Keys. Fly-fishing is not incipient, since the local guides have more than 20 years of experience and current boom of the activity allows a development from the private to foreign sector outside the local GS, with management measures that benefit the community first. Finally, from the analysis, the main threats to the sustainability of the Ascension Bay SES, in the natural system are: hurricanes, storms, and increase sea temperatures; and in the human system: the continuous growth of the demand for tourist services, the ecological impact for coastal development, and the lack of an efficient system for waste disposal. An important aspect is the lack of information on the demographic trend of the community, since more people (local and foreign) demand for jobs.

## Conclusions

It is possible to achieve a holistic understanding of MRSs by applying the Ostrom framework and subdividing the SES based on the different types of resources and users. The holistic conceptual map developed here, adequately illustrated the social-ecological system that shows the interactions of actors with resources, as well as the influence of the GSs in the social performance. This work confirms that governance is an important element to achieve the sustainable use of resources. At the federal level, governance establishes the general rules for users to access or extract resources, while at the local level users can establish more specific rules (collective-choice rules) that allow an equitable distribution of resources, which gives cohesion to the system. However, the GSs in the same community differ from one activity to another. Community-based governance allowed the people of Punta Allen to develop skills and knowledge of natural conservation, which gave to the community a strong sense of appropriation of the resources in the bay. This is because the community feels trust in the federal government which gives it the exclusive rights to fish for lobster or operate the tourist boat trip service. This is not the case of recreational fishing, in which licenses have also been granted for boat services to fishing lodges. The RS could provide greater benefits to the community, particularly in the fly-fishing and ecotourism, but at the cost of greater environmental impacts, which in turn would reduce the capacity or quality of the RS to maintain the economic activities. Likewise, the SESF can be useful as a diagnosis tool to strengthen the local governance and improve the enforcement of environmental regulations.

It is important to continue developing methods to collect information on the 56 second-tier variables, particularly on those that are relevant for the conceptual models. In a context of poor data or lack of first-hand records, interviews and stakeholder surveys can help to obtain qualitative and quantitative information that is very well adapted to the Ostrom SES framework.

## Supplemental Information

10.7717/peerj.7374/supp-1 Supplemental Information 1 Economic analysis of livelihoods.

10.7717/peerj.7374/supp-2 Supplemental Information 2 Tourist cooperative logbooks.

10.7717/peerj.7374/supp-3 Supplemental Information 3 GPS tracks with dataloggers of recreational fishing areas.

10.7717/peerj.7374/supp-4 Supplemental Information 4 Surveys of recreational fishing guides.

10.7717/peerj.7374/supp-5 Supplemental Information 5 Survey fishers lobster raw data.

10.7717/peerj.7374/supp-6 Supplemental Information 6 Survey for anglers.

## Additional Information and Declarations

### Data Availability

The following information was supplied regarding data availability: The raw data are available in Datasets S1–S5. The raw data shows the outputs of surveys, interviews, and logbooks.
